# Applicability of the university of pennsylvania smell identification test (SIT) in brazilians: pilot study

**DOI:** 10.1590/S1808-86942010000600004

**Published:** 2015-10-19

**Authors:** Marco Aurélio Fornazieri, Fábio de Rezende Pinna, Thiago Freire Pinto Bezerra, Marcelo Barros Antunes, Richard Louis Voegels

**Affiliations:** 1 Otorhinolaryngologist; 2 Doctoral degree in otorhinolaryngology, Medical School of the Sao Paulo University (Faculdade de Medicina da Universidade de São Paulo). Assisting physician of the Clinic Hospital of the Sao Paulo University (Hospital das Clínicas da Universidade de São Paulo); 3 Doctoral student in otorhinolaryngology, Medical School of the Sao Paulo University; 4 Otorhinolaryngologist, Department of Otorhinolaryngology - Head and Neck Surgery, University of Pennsylvania; 5 Associate professor, Medical School of the Sao Paulo University (Faculdade de Medicina da Universidade de São Paulo). Division of the Otorhinolaryngology Clinic, Clinic Hospital, Medical School of the Sao Paulo University (Faculdade de Medicina da Universidade de São Paulo)

**Keywords:** olfaction disorders, olfactory perception, smell

## Abstract

The University of Pennsylvania Smell Identification Test (SIT) is the most cited olfactory test in the literature because it is easy to perform and there is high test-retest reliability. There were no standardized olfaction values in a normal Brazilian population.

**Aim:**

To measure the SIT score in a group of Brazilians, and to assess the level of difficulty when implementing the test.

**Study design:**

A cross-sectional study.

**Materials and Methods:**

The SIT was applied in 25 Brazilian volunteers of various income levels who presented no olfactory complaints. Following the test, subjects answered a questionnaire with a visual analog scale (VAS) for the level of difficulty.

**Results:**

The mean in the sample of Brazilians was 32.5 (SD: 3.48) our of 40; this is below what is considered normal for US citizens. The level of difficulty was on average 26 mm (SD: 24.68) in the VAS, but it trended towards easy; 4(16%) participants did not recognize some of the odors under ‘alternatives’.

**Conclusion:**

In this pilot study, there was evidence of good test applicability; the score of the sample of Brazilians was just below normosmia. Further studies are needed to confirm the existence of differences between people of different income levels.

## INTRODUCTION

Although olfaction is paramount for assessing the taste of food and perceiving leaking gases or fire, olfaction evaluation tests have not yet been standardized in Portuguese or in Brazil.

In most cases when olfaction is evaluated, patients are asked to identify odors such as those of coffee, ammonia, chocolate, orange, etc. Such analysis is only part of a qualitative assessment; other criteria are missing, such as the olfactory identification threshold, to separate various levels of loss of olfaction.^1^

The University of Pennsylvania Smell Identification Test (UPSIT or SIT), comprising 40 different odors, is a quick self-administered easily applied test to quantitatively assess human olfaction; it is also has high test-retest reliability (r=0.94).^2^, ^3^, ^4^, ^5^, ^6^, ^7^ Its scores correlate strongly with the traditional olfaction threshold detection test which uses phenyl-ethyl-alcohol.^2^, ^8^ Performance is quite uniform when the SIT is administered in different laboratories using a standard method.^9^

The original test was designed in English; there are currently several translations, including into Portuguese. The test is well accepted and disseminated in English; however, standard SIT scores may be affected by culture, and therefore cannot be generalized. The present study is the first to apply the commercial SIT Portuguese version. The purpose of this pilot study was to verify the difficulty of the test and the scores in a sample of Brazilian subjects, and to check the applicability of the Portuguese version of the SIT in Brazilians.

## MATERIAL AND METHODS

The institutional review board evaluated and approved the study - protocol no. 0359/09.

The UPSIT, commercially known as the Smell Identification TestT (Sensonics Inc., Haddon Hts., NJ 08035), was applied to a group of 25 Brazilian subjects. The study population was chosen randomly from the community, which included income level classes A, B, and C, according to the Brazilian Survey Company Association (Associação Brasileira de Empresas de Pesquisa or ABEP).^10^ No participant had olfactory complaints. Patients with neurological diseases, a history of cranial trauma, upper airway infection on the day of the test, and patients in income levels D and E (ABEP), as explained below, were excluded.

A neurologist and an otorhinolaryngologist (both Brazilian) translated the SIT, supervised by its creator.^5^ The difficulty level of the Portuguese version of the SIT was checked by a questionnaire that was given soon after applying the test. A visual analogue scale (VAS) was used to measure the difficulty in taking the test. This consisted of a 100 mm line with no marks onto which subjects placed a vertical line to indicate the difficulty they encountered when taking the test; easy was toward the left and difficult was towards the right of the line.

The ABEP criteria were used to define income levels; they are used to estimate the purchasing power of urban people and families, taking into account possessions and education to categorize the population into five income levels: A, B, C, D, and E. Class A has the highest purchasing power. Truly and functionally illiterate patients, found mostly in classes D and E, were not included in this study.

**Figure 1:**
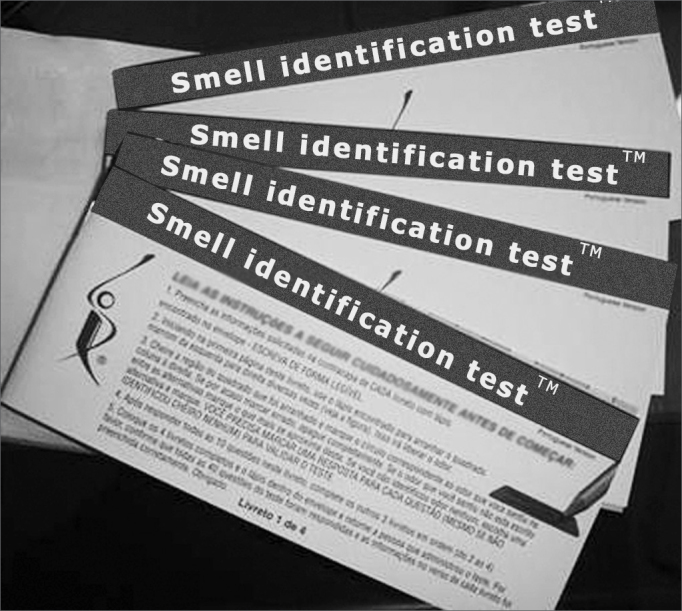
University of Pennsylvania Smell Identification Test (Portuguese version)

**Figure 2:**
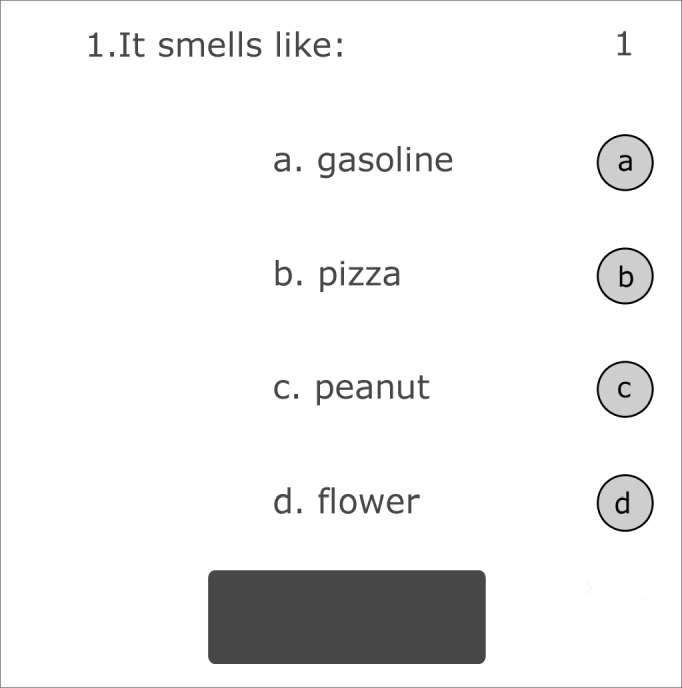
Model of a test page.

The SIT consists of four cards with 10 odors, one per page. Stimuli are contained in plastic microcapsules on a brown strip on the footnote. The examiner asks the patient to scrape the strip with a pencil, which releases the odor. The patient then marks the option that best describes the odor. A score results at the end of the test, which in turn results in a classification as normal olfaction, hyposmia (mild, moderate, severe), and anosmia. (Figs. 1 and 2).

Levene's tests was applied to check the variances, after which a comparison of SIT means was made for each income level using a general linear model to accommodate heteroscedasticity.

## RESULTS

**Chart 1:**
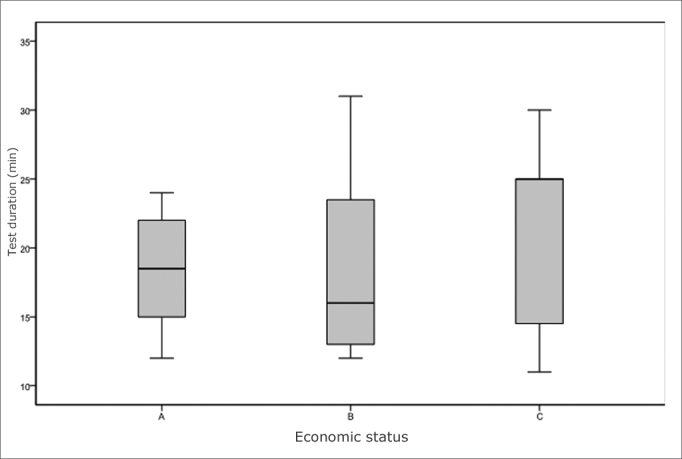
Box plot of the time taken to perform the test according to each income level.

There were 25 patients, 13 (52%) male and 12 (48%) female, aged from 19 to 58 years (mean age - 32.44 years, standard deviation - 11.53 years). One patient smoked. There were 13 (52%) whites, 8 (32%) brown, 4 (16%) yellow, and no black patient. The mean difficulty level was 26 mm (standard deviation - 24.68) according to the VAS; 22 (88%) participants recognized all odors in the alternatives. Odors that some patients did not know were menthol and jasmine. The test time ranged from 15 to 31 minutes (Chart 1). The mean score for the Brazilian sample was 32.5 (standard deviation - 3.48) of 40, which was below the score 34 or more that is considered the normal odor identification score in the US population. Table 2 shows that the poorer scores were for flower (36%), cucumber (36%) and popcorn (24%).

**Table 2:** Percentage of subjects that answer each STI item correctly

Pizza 80%
Peanut 80%
Bubble gum 92%
Rose 88%
Menthol 96%
Natural gas 100%
Cherry 60%
Soap 52%
Engine oil 64%
Garlic 96%
Mint 92%
Grape 100%
Banana 92%
Wood 72%
Clove 100%
Smoke 96%
Leather 96%
Grass 76%
Coconut 92%
Onion 100%
Solvent 84%
Fruit juice 88%
Watermelon 100%
Baby talcum powder 100%
Jasmine 92%
Cinnamon 92%
Gasoline 76%
Strawberry 100%
Coffee 72%
Chocolate 52%
Apple 76%
Flower 36%
Popcorn 24%
Peach 92%
Tire 68%
Cucumber 36%
Pineapple 100%
Raspberry 80%
Orange 100%
Nuts 72%

**Chart 2:**
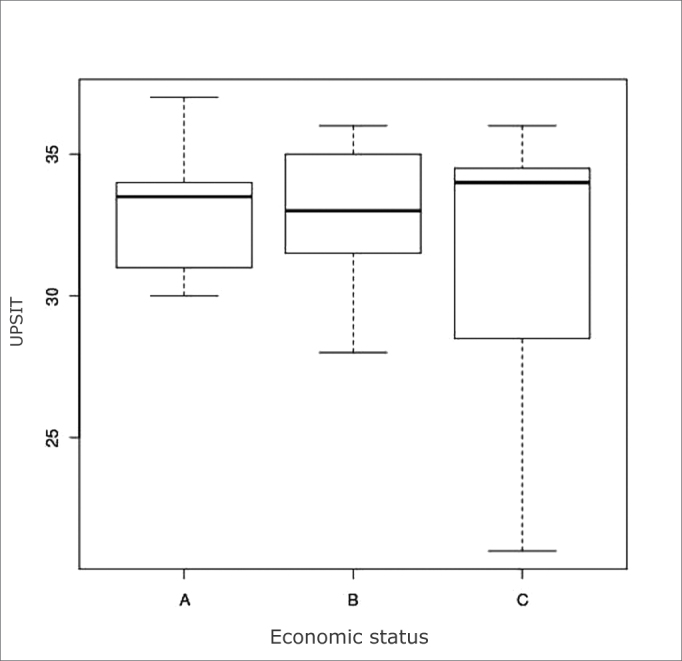
Box plot of the UPSIT score per income level.

There were 6 (24%) income level A subjects, 12 (48%) level B, and 7 (28%) level C (ABEP 2008 classification), shown on Table 1. There was no evidence of different variances at a 5% significance level (p=0.02734), shown on Chart 2.

**Table 1:** Epidemiological data of 25 test volunteers

	Male	Female	Total
Sex	13 (52%)	12 (48%)	25
Age	28 ± 8	38 ± 13	
Income level: A	4 (30.7%)	2 (16.6%)	6 (24%)
B	7 (53.8%)	5 (41.6%)	12 (48%)
C	2 (15.3%)	5 (41.6%)	7 (28%)

## DISCUSSION

The UPSIT is a good choice for accurately measuring olfaction in the clinical practice of Brazilian otorhinolaryngologists. Patients of the three income levels concluded the test easily and in a short time period; the maximum test time was 31 minutes.

The SIT helps solve an issue, namely the choice of odors to be used when assessing olfaction. Few odors stimulate the olfactory nerve only without depolarizing the trigeminal nerve at the same time. This may cause confusion, as patients may interpret trigeminal sensitive stimulation only as olfaction.

Another strong point for this test is that the patient's olfactory score is obtained by comparison with the scores of similar subjects (age and gender) of his or her population.^5^ Additionally, the physician may classify patients as having normal olfaction (normosmia), decreased olfaction (mild, moderate or severe hyposmia), absent olfaction (anosmia), and malingerers.

Standard smell tests are also important because olfaction disorders are the sources of numerous medical and legal complaints and the reason for thousands of medical visits every year.^11^ An advantage of the SIT compared to other olfaction tests in the market is that it may be selfapplied. The Sniffin’ Sticks test, used mainly in Europe, requires a technician to present the smell-releasing pens for patients to identify.^12^

The mean score in our sample was lower compared to the normal value in the US population.^5^ Two factors may explain this finding: the city in which the test were carried out, and a change of some odors in the Portuguese version. Calderón-Garcidueñas found significantly lower SIT score values is subjects from Mexico City, relating such scores to air pollution in that city.^13^ Our study was conducted in São Paulo, where air pollution may also have lowered the score of participants.

Some unfamiliar smells for Brazilians were replaced by other more familiar smells in the Portuguese adaptation of the test. For instance, the typical turpentine smell is replaced by popcorn smell in the Portuguese version. This smell scored poorly (24%) in our sample, and may be one of the causes of the lower mean score.

Strawberry and watermelon smells scored 100%, while the cucumber smell attained 36%. Such discrepant values - not seen in US populations^5^ - may also explain the lower score among Brazilians.

All participants successfully took the test, including subjects of lower income levels, as demonstrated in the VAS. There were no statistically significant differences among subjects of different income levels (A, B, and C - ABEP). The lowest scores were from subjects in the income level C; this, however, may also be due to a small number of subjects.

Further studies with larger samples are needed to verify whether normal values for the US population may be applied in the Portuguese version.

## CONCLUSION

At this point the pilot study indicated that the University of Pennsylvania Smell Identification Test may be applicable; the score of Brazilian volunteers was slightly below the normal range of smell for the US population. Additional studies are needed to confirm score differences among people of different income levels.
